# Item response theory evaluation of the biomedical scale of the Pain Attitudes and Beliefs Scale

**DOI:** 10.1371/journal.pone.0202539

**Published:** 2018-09-12

**Authors:** Alessandro Chiarotto, Annette Bishop, Nadine E. Foster, Kirsty Duncan, Ebenezer Afolabi, Raymond W. Ostelo, Muirne C. S. Paap

**Affiliations:** 1 Department of Epidemiology and Biostatistics, Amsterdam Public Health research institute, VU University Medical Center, Amsterdam, The Netherlands; 2 Department of Health Sciences, Faculty of Science, Amsterdam Movement Sciences research institute, Vrije Universiteit, Amsterdam, The Netherlands; 3 Arthritis Research UK Primary Care Centre, Research Institute for Primary Care and Health Sciences, Keele University, Keele, United Kingdom; 4 Department of Special Needs, Education, and Youth Care, Faculty of Behavioural and Social Sciences, University of Groningen, Groningen, The Netherlands; Qazvin University of Medical Sciences, ISLAMIC REPUBLIC OF IRAN

## Abstract

**Objectives:**

The assessment of health care professionals’ attitudes and beliefs towards musculoskeletal pain is essential because they are key determinants of their clinical practice behaviour. The Pain Attitudes and Beliefs Scale (PABS) biomedical scale evaluates the degree of health professionals’ biomedical orientation towards musculoskeletal pain and was never assessed using item response theory (IRT). This study aimed at assessing the psychometric performance of the 10-item biomedical scale of the PABS scale using IRT.

**Methods:**

Two cross-sectional samples (BeBack, n = 1016; DABS; n = 958) of health care professionals working in the UK were analysed. Mokken scale analysis (nonparametric IRT) and common factor analysis were used to assess dimensionality of the instrument. Parametric IRT was used to assess model fit, item parameters, and local reliability (measurement precision).

**Results:**

Results were largely similar in the two samples and the scale was found to be unidimensional. The graded response model showed adequate fit, covering a broad range of the measured construct in terms of item difficulty. Item 3 showed some misfit but only in the DABS sample. Some items (i.e. 7, 8 and 9) displayed remarkably higher discrimination parameters than others (4, 5 and 10). The scale showed satisfactory measurement precision (reliability > 0.70) between theta values -2 and +3.

**Discussion:**

The 10-item biomedical scale of the PABS displayed adequate psychometric performance in two large samples of health care professionals, and it is suggested to assess group-level professionals degree of biomedical orientation towards musculoskeletal pain.

## Introduction

Musculoskeletal (MSK) pain disorders such as low back pain (LBP), neck pain (NP), and osteoarthritis (OA) are a leading cause of disability globally [1]. Moreover, the financial costs of these disorders represent a considerable burden to health care systems and society [2–5]. Clinical practice guidelines (CPGs) support health care professionals (HCPs) who routinely manage patients with these disorders to deliver best practice care [6–8]. However, HCPs managing patients with MSK pain often fail to follow the recommendations of CPGs and, consequently, deliver sub-optimal care [9, 10]. One key explanation for not following CPGs recommendations is that clinical practice behaviour is strongly related to HCPs’ attitudes and beliefs towards MSK pain [11–14]. Considering the influential role of HCPs’ attitudes and beliefs on clinical practice behaviour [12], and to be able to better target training strategies to those HCPs who do not deliver optimal care, there is a need to have sound measurement instruments with which to assess these variables.

Different self-reported multi-item questionnaires exist to measure HCP attitudes and beliefs towards pain and the most thoroughly tested is the Pain Attitudes and Beliefs Scale (PABS) [15]. This questionnaire was developed and tested in the field of LBP [16, 17], and then adapted for other disorders like NP and OA of the knee [18–20]. The PABS measures the strength of two theoretically derived clinical approaches by means of two subscales: one covering a biomedical approach and one a biopsychosocial approach [16, 17]. Several studies have shown satisfactory Cronbach’s alpha, test-retest reliability, construct validity, structural validity and responsiveness for the biomedical scale, whereas unsatisfactory Cronbach’s alpha and structural validity highlight the need for major reworking of the biopsychosocial scale [15, 21]. The component items recommended for inclusion in the biopsychosocial subscale have varied markedly in previous investigations using the PABS, driven mainly by attempts to improve the dimensionality of the subscale [22–25].

### Item response theory

Item response theory (IRT) provides an excellent framework and toolbox for psychometric evaluations as it encompasses a family of measurement models that focus on explaining the dependencies between item responses within a person and between persons. IRT models are especially suitable for dichotomous or polytomous (e.g. Likert-type scale) item response data [26, 27], like those of the PABS. IRT permits the assessment of dimensionality of a scale and measurement precision at the item level [26, 27]. Some analytic features of IRT cannot be obtained with classical test theory (CTT) analysis, such as item parameters and reliability estimation along the continuum representing the measured latent trait, and examination of the optimal number of response options in each item [26–31]. Reliability estimation of a measurement instrument is usually represented by a single fixed number such as Cronbach’s alpha; yet, this is in conflict with the fact that a scale cannot be expected to measure each person equally efficiently along the latent trait. In IRT, this problem is solved by using (Fisher) information function as an estimate of reliability/measurement precision conditional on the latent trait value; this function, showing information for different latent trait values, is known as scale information function (SIF) [26, 27].

### Aims of the study

IRT methods provide a valid way to assess and refine scales, however, response option sparseness was highlighted as a key finding of the biopsychosocial scale of the PABS [11] and needs to be resolved prior to IRT testing. Exploratory factor analysis (EFA) has shown the 10-item biomedical scale of the PABS to be unidimensional in samples of HCPs from the Netherlands [16]. Nevertheless, some items displayed factor loadings at the lower limit for acceptability and the dimensionality was never assessed in HCPs from other countries, making it crucial to further investigate its psychometric performance in other samples and with other analytic methods. Since the goal of the PABS biomedical scale is to provide adequate information for different degree of biomedical attitudes and beliefs towards pain, an IRT analysis of this scale is warranted. Nevertheless, to date, no studies have assessed the measurement properties of the biomedical scale of PABS with IRT methods. Therefore, this study aimed to use IRT to further assess the psychometric performance of the biomedical scale of the PABS.

## Materials and methods

### Study participants

This study used secondary data analysis of two large samples of HCPs in the UK: one assessing their biomedical orientation towards LBP (BeBack study) [11], and the other towards MSK pain more broadly (DABS study).

The BeBack study was a cross-sectional postal survey of general practitioners (GPs) and physiotherapists (PTs) involved in the management of LBP, conducted between April and November 2005 [11]. This study aimed to explore associations between HCPs attitudes and beliefs towards LBP and their reported clinical behaviour. Simple random sampling was used to obtain details of 2000 GPs and 2000 PTs from national databases [11]. A single reminder was sent to all non-responders four weeks after the first mailing; no incentives were provided for completing the questionnaire [11]. The overall response rate was 38% for a total of 1534 HCPs (443 GPs and 1091 PTs); 66.7% of these (n = 1022, 442 GPs and 580 PTs) reported treating at least one patient with LBP in the previous six months and were included in the analyses of the original study [11].

The DABS dataset used in the current study was a cross-sectional psychometric study involving GPs, PTs, chiropractors and osteopaths. Random samples of HCPs involved in the management of patients with MSK pain (1650 GPs, 750 PTs, 749 chiropractors, 250 osteopaths) were identified through national registries: Binleys (GPs), Chartered Society of Physiotherapy (PTs), British Chiropractic Association (chiropractors), Institute of Osteopathy (osteopaths). A study pack was mailed and contained: letter of invitation, participant information sheet, PABS, and pre-paid return envelope. After two weeks, non-responders were sent a reminder postcard. Two weeks later the study pack was sent again to non-responders and, if a response was not received within two weeks, potential participants were not contacted again. Overall response rates were: 17.7% for GPs, 41.7% for PTs, 45.1% for chiropractors, and 31.6% for osteopaths. After selecting only professionals that treated patients with LBP in the previous six months: 279 GPs, 268 PTs, 329 chiropractors and 78 osteopaths were included.

Ethical approval for the BeBack study was obtained from the West Midlands Multi-centre Research Ethics Committee (MREC) (reference 05/MRE07/1), and for the DABS study from Keele University Ethics Review Panel.

### Measurement instrument

The PABS was developed to measure PTs’ attitudes and beliefs about non-specific LBP to determine the degree to which they adopted a biomedical or a biopsychosocial treatment approach [17]. The two-factor structure of the original scale was in line with the intentions of the developers [17], however, the number of items in each subscale was reduced by means of EFA into a 19-item version in a subsequent study [16]. Each PABS item is rated on a 6-point Likert scale, ranging from ‘Totally disagree’ (score = 1) to ‘Totally agree’ (score = 6). Ten items load on one subscale representing the biomedical orientation (total score range: 10–60), while the other nine load on the biopsychosocial subscale (total score: 9–54). This version of the PABS was developed and refined in PTs in the Netherlands and two of its items were slightly amended in the version used in the UK, to ensure face validity for both GPs and PTs [11]. Also in a sample of PTs and GPs in the UK a two-factor structure was found [25]. Considering its satisfactory measurement properties, the 10-item biomedical scale was retained in the DABS study in which a new MSK generic version of the PABS was developed to measure HCPs’ attitudes and beliefs towards MSK pain more broadly. Small amendments were made in five items (i.e. 2, 3, 4, 5 and 10) of the PABS biomedical scale to make it applicable to different MSK pain conditions.

### Statistical analysis

All analyses were performed in the two datasets (BeBack and DABS) separately. The following three main steps were undertaken in the analyses: 1) missing data handling and descriptive statistics, 2) evaluation of IRT assumptions, 3) IRT fit evaluation and estimations.

### Missing data handling and descriptive statistics

Frequencies of missing data at the item level was calculated and respondents with missing data on all items of the scale were excluded from analysis. Patterns of missing values were explored to find any recurrent pattern. To evaluate if a desirable ‘missing completely at random’ (MCAR) situation was present, the Little MCAR’s test was used, with a cut-off p-value > 0.05 [32]. If less than 10% of respondents displayed missing data and data were MCAR, a two-way imputation technique was used at the item level [33–35].

Response frequencies for each category of each item were also assessed. If fewer than 10 participants endorsed a response option, that option was collapsed with the contiguous one that had a similar meaning. Descriptive statistics were calculated for the socio-demographic characteristics of the participants. All descriptive statistics and missing data handling were conducted with the statistical software SPSS, version 21.

### Evaluation of dimensionality and local independence

Following Lenferink et al. [36], we used two complementary statistical methods to evaluate the dimensionality of the PABS: 1) common factor analysis, 2) Mokken scale analysis (MSA; a non-parametric technique). Factor analysis was performed using the software programme FACTOR [37] (version 10.8.01), and MSA using the R package Mokken version 2.8.10 [38].

The procedure used for determining the number of factors was Parallel Analysis based on Minimum Rank Factor Analysis; this method will be abbreviated as PA-MRFA [39]. PA-MRFA can be seen as the current gold standard method for exploratory factor analysis. In PA-MRFA the empirical value of the proportion of explained common variance (ECV) is compared to corresponding factors ECV derived from random data [39]; this is done for each factor separately. The random data are generated based on the sample size of the real data assuming independence among items [40]. To determine the optimal number of factors, the observed ECV associated with a factor can be compared to the mean or the 95^th^ percentile of the sampling distribution associated with the corresponding factor. We used the standard configuration for PA-MRFA available in FACTOR: 500 random correlation matrices were generated based on “random permutation of sample values” [39]. The factor analyses were based on the polychoric correlation matrix.

MSA investigates the dimensionality of a set of items and, at the same time, identifies scales that allow an ordering of respondents on one or more underlying one-dimensional scales using the unweighted sum of item scores [41–43]. The imputed dataset was used as MSA is not appropriate for use with missing data [44]. Scalability coefficients (denoted as *H*) are calculated on several levels (scale: *H*; item: *H*_*i*_; item-pair: *H*_*ij*_). *H*_*ij*_ and *H*_*i*_-values can be used to determine which of the items form a scale; the *H*_*i*_-value expresses the degree to which an item is related to other items. The *H* coefficient expresses the degree to which the total score can be reliably used to order respondents on the latent trait. A scale is considered acceptable if 0.3 ≤ *H* < 0.4, good if 0.4 ≤ *H* < 0.5, and strong if *H* ≥ 0.5 [42]. First, a confirmatory analysis was run and an *H* ≥ 0.3 for the total scale was considered satisfactory [41, 42]. Second, an exploratory analysis was performed using the Automated Item Selection Procedure (AISP). The AISP is a bottom-up, iterative approach in which a starting pair of items is selected with a favourable *H*_*ij*_ value, after which one item at a time is added to form a scale. Items are only added to the scale if they have a positive relationship (*H*_*ij*_) with the other items in the scale, and if the selected item has an *H*_*i*_-value exceeding a pre-defined lowerbound. This analysis included successive iterations in which the lowerbound scalability coefficient was increased by 0.1, from 0.1 to 0.5. The resulting pattern of outcomes is thought to be indicative of the dimensionality of a set of items. The scale was assumed to be unidimensional if at lowerbounds from 0.1 to 0.3, only one scale, or a bigger scale and a smaller one, were found [42].

Local independence signifies that, after controlling for the dominant construct, there should not be residual correlations among items [26–29, 31, 45]. Local independence was also assessed under MSA, using the R package *Mokken* version 2.8.10 [38].

### IRT fit evaluation and estimations

For model fit, we estimated the 1PL, the GPCM and the 2PL Samejima’s Graded Response Model (GRM) in the R package *mirt* version 1.26.3 [46], to ascertain which of these models showed the best overall fit. Original datasets, not imputed, were used for parametric IRT models as they can handle the presence of missing data [47]. The Akaike Information Criterion (AIC) was used to determine which model provided the best fit to the data [48]. The AIC allows comparison of non-nested models when the parameters within the models are estimated by the method of maximum likelihood and identifies the most parsimonious model by taking into account both goodness of fit and complexity of the models [48, 49]. In this study, the GRM was the model with the best data fit.

Model fit was assessed with S-X^2^ item fit statistics for polytomous data, which quantify differences between observed and expected response frequencies under the GRM model [50, 51]. S-X^2^ statistics with a p-value <0.001 were considered to indicate item misfit [45].

Among item parameters, item thresholds (β, or item difficulty parameters) represent the level of difficulty of an item and its response options, and item slopes (α, or item discrimination parameters) indicate the relationship of an item with the measured construct with higher values indicating a greater ability of an item to discriminate between adjoining values on the construct [26–29, 31, 45].

Item characteristic curves (ICCs) and item information functions (IIFs) were estimated for each item under the GRM. ICCs illustrate visually the probability of selecting the response options of an item considering the level of a respondent on the estimated underlying theta [26–29, 31, 45]. IIFs were estimated to determine which items were the most precise in measuring different levels of theta [26, 27, 31, 45]. All IIFs were summed to plot a SIF that gives an indication of the measurement precision of the total scale across different levels of the latent trait [26, 27, 31, 45]. In this context, information is conceptualized as an index of local reliability (r), where r can be calculated as 1-(1/information) to obtain a 0–1 value [30, 31]. A value of r > 0.70 is usually used to consider an instrument as having satisfactory reliability when comparing population means [52–54] and this value corresponds to information > 3.3 [30, 31]. A standard error (SE) of the estimated theta can also be calculated, being the inverse of the square root of information [28, 31, 45]. ICCs displayed items with response options having an endorsement probability lower than contiguous options, analyses were repeated after collapsing these response categories to assess if this led to an improvement in unidimensionality and measurement precision of the scale. All IRT parametric analyses were conducted using the R package *mirt* version 1.26.3 [46]. The GRM was estimated using a full information maximum likelihood approach.

## Results

### BeBack study

Six participants had missing data on all items and were excluded from analysis leaving a total sample of 1016 respondents. Analysis of missing data revealed that 56 participants (5.5%) had at least one missing item and that 67 item values (0.7%) were missing in total. The Little MCAR’s test was not significant (*p* = 0.968) suggesting that missing were completely at random.

Descriptive statistics for the socio-demographic characteristics of the HCPs are presented in Table 1, while descriptive statistics for the 10 items of the scale are displayed in Table 2. The sample had a mean score of 31.0 (standard deviation (SD) = 6.5) on the scale. The lowerbound of the reliability, estimated using Cronbach’s alpha, equalled 0.78.

**Table 1:** Socio-demographic characteristics of health care professionals included in the two samples used in this study.

	BeBack (*n* = 1016)	DABS (*n* = 958)
Clinical profession, *n* (%)		
General practitioners	439 (43.2%)	279 (29.1%)
Physiotherapists	577 (56.8%)	268 (28.0%)
Chiropractors	0 (0.0%)	329 (34.3%)
Osteopaths	0 (0.0%)	78 (8.1%)
Missing information	0 (0.0%)	4 (0.4%)
Gender, *n* (%)		
Male	364 (35.8%)	415 (43.3%)
Female	643 (63.3%)	532 (55.5%)
Missing information	9 (0.9%)	11 (1.1%)
Years from professional qualification, mean (SD)*	20.7 (10.7)	19.1 (10.6)
Postgraduate MSK Training, *n* (%)		
Yes	497 (48.9%)	595 (62.1%)
No	504 (49.6%)	347 (36.2%)
Missing information	15 (1.5%)	16 (1.7%)
Clinical specialty, *n* (%)		
Yes	590 (58.1%)	377 (39.4%)
No	410 (40.4%)	546 (57.0%)
Missing information	16 (1.6%)	35 (3.7%)
Presence of LBP in the past, *n* (%)		
Yes	717 (70.6%)	/
No	281 (27.7%)	/
Missing information	18 (1.8%)	/
Proportion of work in clinical practice, *n* (%)		
76–100%	/	825 (86.1%)
50–75%	/	96 (10.0%)
<50%	/	32 (3.3%)
Missing information	/	5 (0.5%)
Work setting, *n* (%)		
Exclusively in the NHS	/	360 (37.6%)
Exclusively in non-NHS	/	479 (50.0%)
Combination of NHS and non-NHS	/	114 (11.9%)
Missing information	/	5 (0.5%)
Proportion of patients seen with MSK disorders, mean (SD)**	/	53.8 (34.4)

MSK = musculoskeletal; LBP = low back pain; NHS = National Health Service (in United Kingdom).*n* = number; % = percentage on the total; SD = standard deviation; / = not assessed.

*Data on this variable were missing for 52 respondents in BeBack and for 54 in DABS.

**Data on this variable were missing for 54 respondents in DABS.

**Table 2:** Descriptive statistics for the 10 items of the biomedical scale of the Pain Attitudes and Beliefs Scale (PABS).

	Score range	Mean (SD)	Skewness (SE)	Kurtosis (SE)	Item-total correlation	Cronbach’s alpha if item deleted
BeBack (*n* = 1016)						
Item 1 –Pain is a nociceptive stimulus, indicating tissue damage	1–6	3.5 (1.1)	-0.30 (0.08)	-0.36 (0.15)	0.445	0.76
Item 2 –Patients with back pain should preferably practice only pain free movements	1–6	2.8 (1.1)	0.54 (0.08)	-0.13 (0.15)	0.394	0.77
Item 3 –Back pain indicates the presence of organic injury	1–5*	2.8 (1.1)	0.11 (0.08)	-0.68 (0.15)	0.438	0.76
Item 4 –If back pain increases in severity, I immediately adjust the intensity of treatment	1–6	3.7 (1.2)	-0.06 (0.08)	-0.43 (0.15)	0.352	0.77
Item 5 –If treatment does not result in a reduction in back pain, there is a high risk of severe restrictions in the long term	1–6	3.4 (1.1)	-0.10 (0.08)	-0.67 (0.15)	0.326	0.77
Item 6 –Pain reduction is a precondition for the restoration of normal functioning	1–6	3.7 (1.2)	-0.31 (0.08)	-0.68 (0.15)	0.493	0.75
Item 7 –Increased pain indicates new tissue damage or the spread of existing damage	1–5*	2.7 (1.0)	0.31 (0.08)	-0.40 (0.15)	0.618	0.74
Item 8 –If patients complain of pain during exercise, I worry that damage is being caused	1–5*	2.7 (1.0)	0.26 (0.08)	-0.48 (0.15)	0.582	0.74
Item 9 –The severity of tissue damage determines the level of pain	1–5*	2.4 (1.1)	0.64 (0.08)	-0.28 (0.15)	0.520	0.75
Item 10 –In the long run, patients with back pain have a higher risk of developing spinal impairments	1–6	3.1 (1.1)	0.14 (0.08)	-0.75 (0.15)	0.324	0.77
DABS (*n* = 958)						
Item 1 –Pain is a nociceptive stimulus, indicating tissue damage	1–6	3.6 (1.2)	-0.37 (0.08)	-0.55 (0.16)	0.505	0.75
Item 2 –Patients with musculoskeletal pain should preferably practice only pain free movements	1–5*	2.9 (1.1)	0.27 (0.08)	-0.47 (0.16)	0.492	0.75
Item 3 –Musculoskeletal pain indicates the presence of organic injury	1–5*	3.0 (1.1)	0.07 (0.08)	-0.63 (0.16)	0.473	0.75
Item 4 –If pain increases in severity, I immediately adjust the intensity of treatment	1–6	4.2 (1.1)	-0.34 (0.08)	-0.15 (0.16)	0.304	0.77
Item 5 –If treatment does not result in a reduction in pain, there is a high risk of severe restrictions in the long term	1–6	3.7 (1.1)	-0.27 (0.08)	-0.30 (0.16)	0.325	0.77
Item 6 –Pain reduction is a precondition for the restoration of normal functioning	1–6	4.1 (1.1)	-0.60 (0.08)	-0.10 (0.16)	0.378	0.76
Item 7 –Increased pain indicates new tissue damage or the spread of existing damage	1–5*	2.8 (1.1)	0.41 (0.08)	-0.26 (0.16)	0.632	0.73
Item 8 –If patients complain of pain during exercise, I worry that damage is being caused	1–5*	2.9 (1.1)	0.24 (0.08)	-0.77 (0.16)	0.540	0.74
Item 9 –The severity of tissue damage determines the level of pain	1–5*	2.5 (1.2)	0.48 (0.08)	-0.41 (0.16)	0.561	0.74
Item 10 –In the long run, patients with musculoskeletal pain have a higher risk of developing functional impairments	1–6	4.0 (1.3)	-0.56 (0.08)	-0.48 (0.16)	0.263	0.78

SD = standard deviation; SE = standard error; *n* = number

*The response categories ‘totally agree’ and ‘largely agree’ were merged for these items (see explanation in the text).

The factor analysis showed support for a unidimensional solution. The polychoric correlation matrix can be found in S1 Table. Only the first factor explained a larger percentage of common variance (69.3%) that could be expected when using random data (mean: 33.3%, 95^th^ percentile: 47.4%); the second factor explained a smaller percentage of common variance (11.3%) that expected when using random data (mean: 26.1%, 95^th^ percentile: 35.0%). In contrast, an *H* value of 0.273 was found for the total scale using confirmatory MSA, which is below the threshold of 0.3 for an acceptable scale. *H*_*i*_ values for the individual items are presented in Table 3. The results of running exploratory analyses for increasing values of lowerbound scalability coefficient were inconclusive. At lowerbounds 0.1 and 0.2 all ten items were placed in the first scale, while at lowerbound 0.3 five items (i.e. 2, 6, 7, 8, 9) were placed in the first scale, two items (1, 3) in a second smaller scale, the other three items (4, 5, 10) were discarded. The *H* value of the first and largest scale was equal to 0.4. No locally dependent item pairs were found under MSA. Since the FA showed support for a unidimensional solution, IRT analyses were performed using unidimensional models.

**Table 3:** Results of item response theory analysis, including scalability coefficients, item fit statistics, and item parameters for the 10 items of the biomedical scale of the Pain Attitudes and Beliefs Scale (PABS).

	MSA H_i_	S-X^2^	p-value S-X^2^	α	β1	β2	β3	β4	β5
BeBack (*n* = 1016)									
Item 1	0.257	104.497	0.158	1.076	-3.336	-1.594	-0.223	1.671	4.211
Item 2	0.244	123.924	0.004	1.029	-2.714	-0.328	1.293	2.512	4.552
Item 3	0.259	90.790	0.287	1.075	-2.163	-0.372	1.188	3.123	/
Item 4	0.222	122.820	0.113	0.807	-4.847	-2.403	-0.477	1.448	3.508
Item 5	0.204	102.813	0.251	0.719	-5.014	-1.822	-0.011	2.249	5.908
Item 6	0.304	119.913	0.023	1.259	-3.389	-1.432	-0.387	0.890	3.158
Item 7	0.369	80.860	0.045	2.452	-1.506	-0.143	0.919	1.863	/
Item 8	0.349	75.481	0.176	1.956	-1.666	-0.097	1.018	2.346	/
Item 9	0.325	93.763	0.025	1.731	-1.140	0.466	1.285	2.466	/
Item 10	0.209	99.163	0.157	0.749	-4.144	-0.809	0.572	3.121	6.137
DABS (*n* = 958)									
Item 1	0.313	92.861	0.341	1.640	-2.337	-1.116	-0.313	1.006	2.815
Item 2	0.297	65.184	0.900	1.244	-2.255	-0.425	0.994	2.363	/
Item 3	0.287	129.931	<0.001	1.382	-1.953	-0.583	0.639	2.118	/
Item 4	0.194	117.774	0.036	0.644	-7.076	-4.436	-1.985	0.584	3.029
Item 5	0.198	123.035	0.138	0.584	-6.438	-2.916	-0.786	2.367	6.182
Item 6	0.246	108.498	0.130	0.784	-5.484	-2.972	-1.470	0.474	3.674
Item 7	0.380	50.165	0.813	2.421	-1.589	-0.179	0.807	1.790	/
Item 8	0.328	69.449	0.563	1.751	-1.924	-0.234	0.621	1.944	/
Item 9	0.347	96.614	0.023	1.766	-1.104	0.147	1.078	2.275	/
Item 10	0.162	145.038	0.040	0.438	-7.488	-3.726	-2.026	0.685	5.370

MSA H_i_ = Mokken scale analysis scalability coefficient; S-X^2^ = item fit statistics under the graded response model; α = Item Discrimination Parameters estimated under the graded response model; β = Item Difficulty Parameters estimated under the graded response model. *n* = number; / = not applicable.

All items exhibited satisfactory item fit statistics under the GRM model (S-X^2^ p-values > 0.001, Table 3). Item thresholds and item slopes estimated are listed in Table 3. Items 4, 5 and 10 were those with the lowest discriminative power and with difficulty parameters covering a larger range of theta values; items 7, 8 and 9 showed the highest discrimination and difficulty covering a smaller range of theta values.

**Fig 1:**
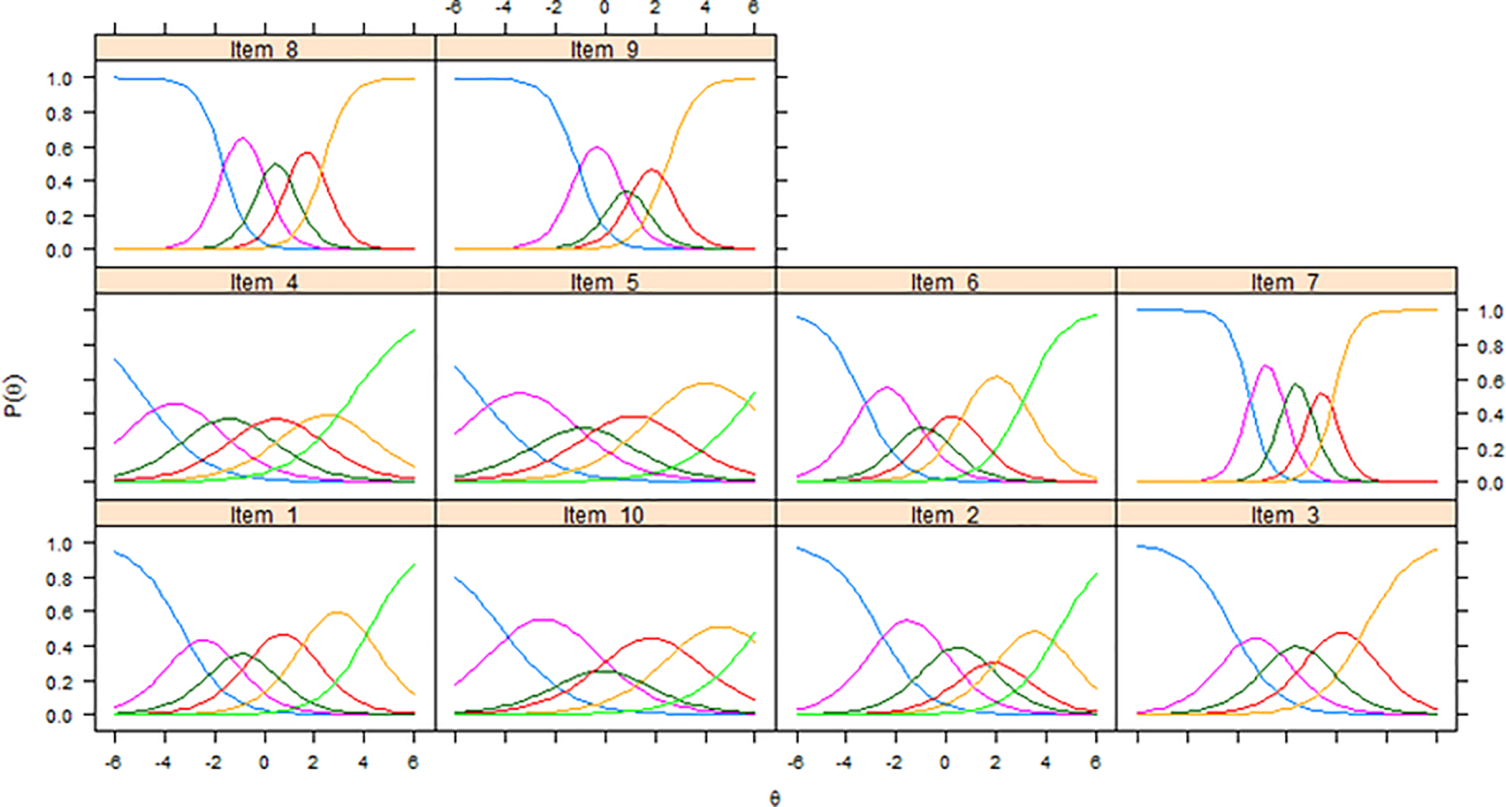
Item characteristic curves of the 10 items of the biomedical scale of the Pain Attitudes and Beliefs Scale (PABS) in the BeBack study.

**Fig 2:**
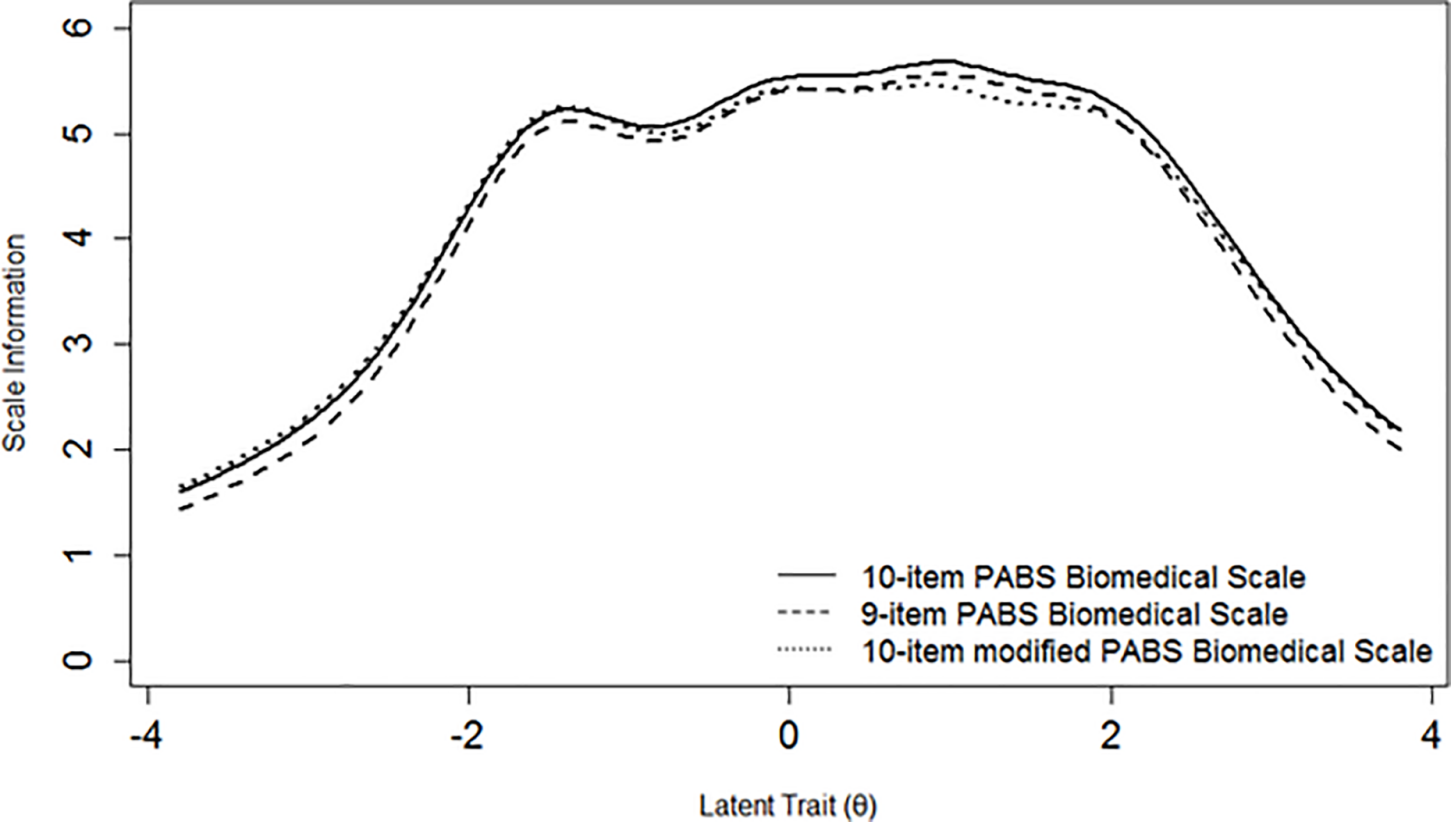
Scale information functions for three versions of the biomedical scale of the Pain Attitudes and Beliefs Scale (PABS) in the BeBack study.

Fig 1 shows ICCs for all items of the scale, while Fig 2 shows the SIF which exhibits acceptable local reliability (i.e. information > 3.3 = r > 0.70) approximately between -2 and +3 theta values.

We decided to rerun all analyses after having removed item 10 as it was the one showing the most poorly psychometric performance (Table 3, Fig 1). This deletion did not lead to any improvement in item slopes or SIF (Fig 2).

All poorly endorsed response options (Fig 1) were merged with adjacent ones having similar meaning (e.g. ‘disagree to some extent’ with ‘agree to some extent’) resulting in a modified 10-item version with varying number of response options across items. All analyses were also rerun for this modified 10-item version. Parametric IRT item parameters did not change substantially and no substantial changes could be identified in the SIF (Fig 2).

### DABS study

All 958 respondents were included in the analyses. Analysis of missing data revealed that 53 subjects (5.5%) had at least one missing item and that 70 item values (0.7%) were missing in total; a MCAR situation was present (Little MCAR’s test,*p* = 0.356).

Table 1 and Table 2 present also the socio-demographic characteristics of the HCPs and item level statistics in this sample. The sample mean score on the scale was 33.7 ± 6.7 SD, its Cronbach’s alpha equalled 0.78.

PA-MRFA exhibited strong support for a unidimensional solution. The polychoric correlation matrix is included in S2 Table. Only the first factor accounted for a larger percentage of common variance (64.4%) than what could be expected when using random data (mean: 33.1%, 95^th^ percentile: 46.4%); the second factor accounted for a smaller proportion (13.2%) than what could be expected with random data (mean: 26.5%; 95^th^ percentile: 34.9%). As for the BeBack sample, the scale scalability coefficient (*H* = 0.274) was below the threshold to be considered an acceptable scale. The three items (4, 5 and 10) found with the lowest *H*_*i*_ values in this dataset were the same as those in the BeBack sample (Table 3). The scale also demonstrated satisfactory unidimensionality: all ten items were assigned to the first scale at lowerbound 0.1, eight items were assigned to the first scale and the other two to a second scale at lowerbound 0.2, six items were assigned to the first scale and four discarded at lowerbound 0.3. At this latter lowerbound, the scale *H* value was 0.426. Local independence assessment did not show any locally dependent item pairs. Since both PA-MRFA and MSA displayed support for a unidimensional solution, IRT analyses were performed using unidimensional models.

**Fig 3:**
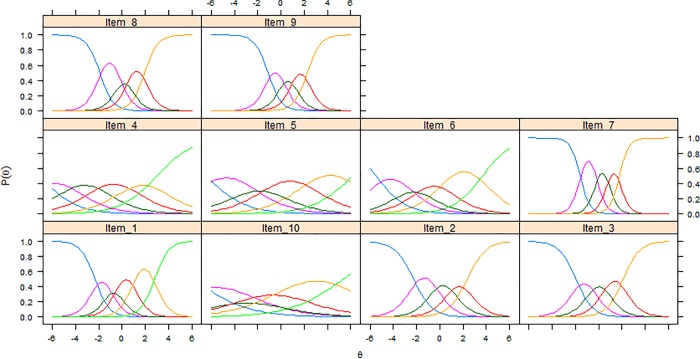
Item characteristic curves of the 10 items of the biomedical scale of the Pain Attitudes and Beliefs Scale (PABS) in the DABS study.

**Fig 4:**
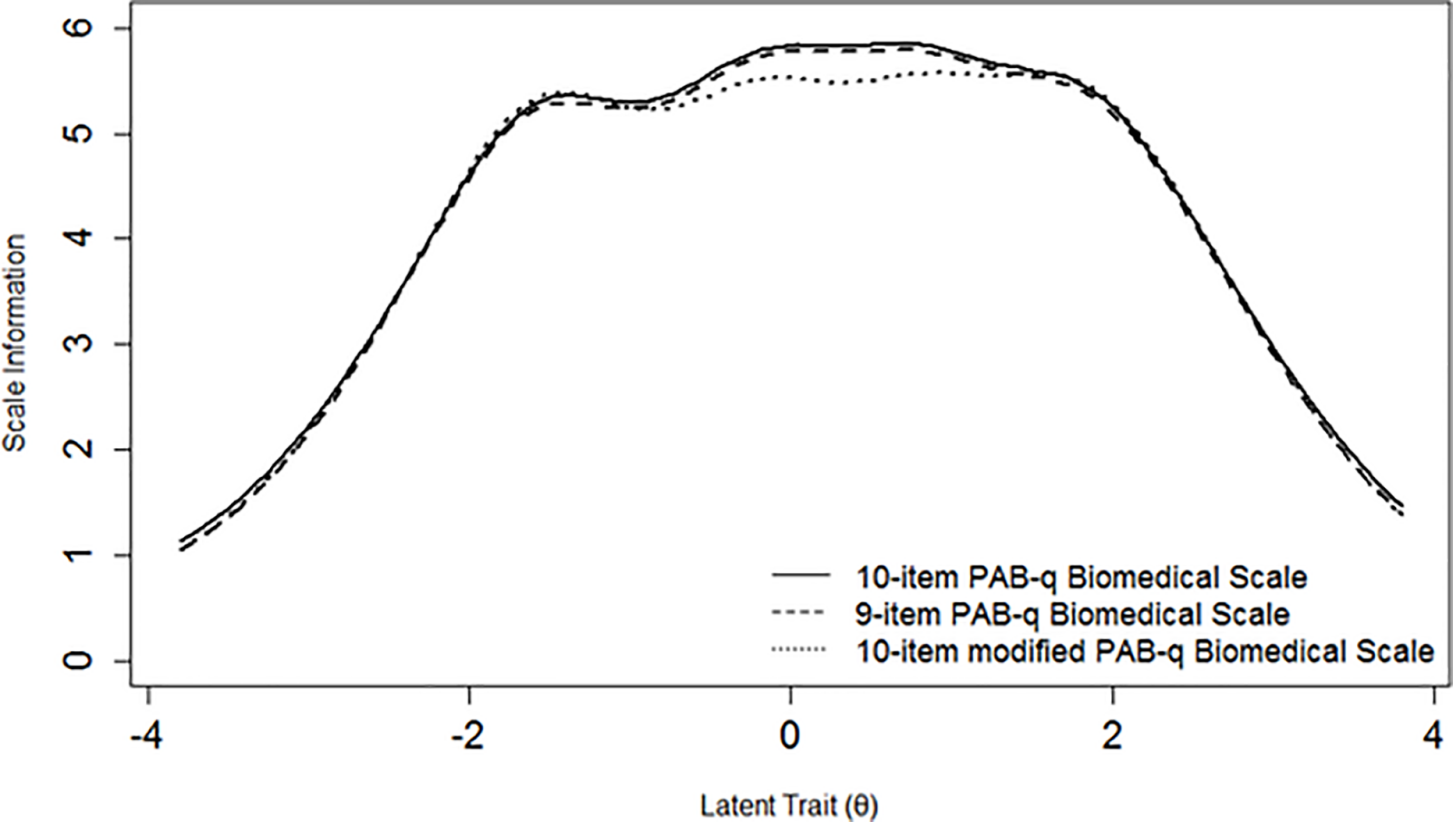
Scale information functions for three versions of the biomedical scale of the Pain Attitudes and Beliefs Scale (PABS) in the DABS study.

Item 3 displayed an unsatisfactory fit statistic (S-X^2^ p-value < 0.001), while all other items fitted the GRM model (Table 3). As in the BeBack dataset, items 7, 8 and 9 were those with highest item slopes, while items 4, 5 and 10 were those with the lowest ones (Table 3); these latter three items together with item 6 were also those with threshold parameters spreading across a broader range of the latent trait (Table 3). Fig 3 displays all ICCs of the biomedical scale PABS version. Also in this sample, the scale exhibited acceptable measurement precision between -2 and +3 theta values (Fig 4).

An additional analysis was run to evaluate if the removal of the worst performing item 10 (consistently with the BeBack dataset) led to substantial improvements in the scale. The SIF of the 9-item version of the questionnaire was very similar to the curve of the original 10-item version (Fig 4).

Analyses were repeated as for the BeBack dataset for a 10-item modified version in which all response options with low probabilities of endorsement were collapsed (Fig 3). No substantial improvement could be observed in item thresholds and item slopes. A loss in information could be observed for theta values between -1 and 2 but without compromising local reliability (Fig 4).

## Discussion

The biomedical scale of the PABS was assessed with IRT analytic methods in two large samples of HCPs in the UK. Factor analyses offered clear support for unidimensionality of the PABS scale in both samples. This finding was supported by the MSA for the DABS sample as well; for the BeBack sample, the MSA findings were inconclusive. Three items (i.e. 4, 5 and 10) were consistently found to show poor discrimination values, and three items (i.e. 7, 8 and 9) showed the highest discrimination, as estimated using the GRM (parameteric IRT). The scale showed satisfactory measurement precision for estimated latent trait values for an acceptable interval around the population mean level.

The PABS was developed following a CTT approach and this is the first study that assesses its biomedical scale with IRT analytic methods. Modern IRT techniques provide some advantages over CTT, providing a deeper insight into the measurement properties of a self-reported questionnaire and its items [26–31]. Our results were very similar in two different samples of HCPs in the UK, one including only GPs and PTs, the other also chiropractors and osteopaths (Table 1). These results are relevant considering that the PABS biomedical scale was originally developed to evaluate PTs’ attitudes and beliefs towards non-specific LBP [17] and subsequently adapted to also assess GPs’ attitudes and beliefs [11]. The same scale, with some small adaptations, was recently included in a new generic MSK version of the PABS to measure attitudes and beliefs of PTs, GPs, chiropractors and osteopaths towards non-specific MSK more broadly. The fact that the questionnaire showed consistently similar results in two different versions and in different HCPs shows that this scale has the potential to be adapted to different MSK pain conditions and HCP populations.

In this study, some issues were consistently identified for items 4, 5 and 10 of the scale in both samples. These items were those with lower MSA scalability coefficients and IRT discrimination parameters (Table 3). These findings are not surprising for item 10 considering that previous EFA studies have shown this item to be the most problematic [16, 17]. Nevertheless, the results for items 4 and 5 in the present study has not been previously reported. The content of items 4, 5 and 10 seem to refer to aspects of the treatment or prognosis of patients with musculoskeletal pain, whereas the items with the highest discriminative power (i.e. 7, 8, 9) refer more to aspects of pain neurophysiology; a similar distinction can also be made with other items (e.g. 1 and 3) that showed acceptable and higher levels of discrimination (Table 2). This apparent difference in content could explain why some items present such low discrimination. These considerations could be further explored in future studies involving experts in the field of pain attitudes and beliefs and asking them to accurately judge the content validity of this scale.

Additional analyses without item 10 indicated that removing this item did not lead to loss of measurement precision (Figs 2 and 4). Considering that this questionnaire has been used in different languages and with reference to different MSK pain conditions [11, 16, 18–20, 23], it seems inappropriate to suggest the removal of item 10 as this would also lead to a discrepancy with the version of the questionnaire used in previous studies. However, if the results of this and prior studies are replicated in other samples, the future removal of item 10 and/or refinement of the scale should be further discussed and reconsidered. The misfit of item 3 in the DABS sample was a new and surprising finding, considering that this item seems to cover a pain neurophysiological aspect, in line with items 7, 8 and 9 which are the best performing ones. Additionally, its discrimination and difficulty parameters were very similar in the two samples (Table 3). For these reasons, we decided of not running additional analyses with the removal of this item.

ICCs of different PABS versions showed that some response options of some items had a low probability of endorsement compared to adjacent options (Figs 1 and 3). We decided to run additional analyses to assess if merging these response options led to positive changes in item parameters and measurement precision. No loss in measurement precision was retrieved (Figs 2 and 4), therefore our findings were not sufficient to justify the merging of these response options as this would lead to an impractical version of the scale with items having varying numbers of response options. Hence, our analyses and considerations are in favor of keeping the PABS biomedical scale in its current form.

The original version of the PABS was developed and tested in the Dutch language and culture [16, 17]. The versions used in this study of HCPs in the UK are an adaptation of the 19-item version refined by Houben et al. [11, 16]. To date, no studies assessing the cross-cultural validity of this questionnaire have been performed. A commonly used definition of cross-cultural validity is ‘the degree to which the performance of the items on a translated or culturally adapted instrument is an adequate reflection of the performance of the items of the original version of the instrument’ [55]. This measurement property can be tested by assessing differential item functioning under an IRT model, for which samples of different language versions should be aggregated [27, 28]. Therefore, considering that this questionnaire is already available in several languages, future international collaborations and studies should attempt to assess this measurement property by merging datasets from different countries.

The results of this and previous studies on the PABS biomedical scale have shown that this scale has exhibited acceptable psychometric performance and precision for group-level analyses of the degree of HCP biomedical orientation towards MSK pain. Future research efforts should be directed towards improving the measurement precision of this scale for individual-level analyses (i.e. to reach reliability estimates ≥ 0.9); this could be accomplished by adding more items that reflect the same construct. Importantly, the original intention of the PABS developers was to have a questionnaire that could classify HCPs as having a biomedical approach or a biopsychosocial approach [17]. In fact, the PABS includes another subscale aimed at assessing the biopsychosocial orientation of HCPs towards pain [11, 16, 17]. This scale was not assessed in the current study because previous research has indicated that it needs psychometric improvement [15, 21].

This discrepancy in the scales’ psychometric performance could be due to different factors, one of them being the widespread diffusion and acceptance of the biopsychosocial model for explaining MSK pain disorders, like LBP [56–59]. In fact, the popularity of this model has probably influenced HCPs’ attitudes and beliefs towards MSK pain, so that it has become difficult for them to ‘disagree’ with items on the biopsychosocial orientation and this has led to sparseness and lack of variation in responses on this subscale. Overall, taking into account the psychometric differences in the two subscales, it can be asserted that research in the field of measurement of attitudes and beliefs towards pain is still at a preliminary stage, and that further psychometric research is necessary.

## Supporting information

S1 Table Polychoric correlation matrix of the biomedical scale of the Pain Attitudes and Beliefs Scale (PABS) in the BeBack data (n = 1016). (DOCX)

S2 Table Polychoric correlation matrix of the biomedical scale of the Pain Attitudes and Beliefs Scale (PABS) in the DABS data (n = 958). (DOCX)

## References

[1] Vos T, Flaxman AD, Naghavi M, Lozano R, Michaud C, Ezzati M, et al Years lived with disability (YLDs) for 1160 sequelae of 289 diseases and injuries 1990–2010: a systematic analysis for the Global Burden of Disease Study 2010. The Lancet. 2013;380(9859):2163–96. 10.1016/S0140-6736(12)61729-2 PMC6350784 23245607

[2] Borghouts JA, Koes BW, Vondeling H, Bouter LM. Cost-of-illness of neck pain in The Netherlands in 1996. Pain. 1999;80(3):629–36. 10342424 10.1016/S0304-3959(98)00268-1

[3] Dagenais S, Caro J, Haldeman S. A systematic review of low back pain cost of illness studies in the United States and internationally. The spine journal. 2008;8(1):8–20. 10.1016/j.spinee.2007.10.005 18164449

[4] Maniadakis N, Gray A. The economic burden of back pain in the UK. Pain. 2000;84(1):95–103. 10601677 10.1016/S0304-3959(99)00187-6

[5] Ruiz D, Koenig L, Dall TM, Gallo P, Narzikul A, Parvizi J, et al The direct and indirect costs to society of treatment for end-stage knee osteoarthritis. The Journal of Bone & Joint Surgery. 2013;95(16):1473–80. 23965697 10.2106/JBJS.L.01488

[6] Childs JD, Cleland JA, Elliott JM, Teyhen DS, Wainner RS, Whitman JM, et al Neck pain: clinical practice guidelines linked to the International Classification of Functioning, Disability, and Health from the Orthopaedic Section of the American Physical Therapy Association. Journal of Orthopaedic & Sports Physical Therapy. 2008;38(9):A1–A34. 10.2519/jospt.2008.0303 18758050

[7] Koes BW, van Tulder M, Lin C-WC, Macedo LG, McAuley J, Maher C. An updated overview of clinical guidelines for the management of non-specific low back pain in primary care. European Spine Journal. 2010;19(12):2075–94. 10.1007/s00586-010-1502-y 20602122 PMC2997201

[8] Zhang W, Moskowitz R, Nuki G, Abramson S, Altman R, Arden N, et al OARSI recommendations for the management of hip and knee osteoarthritis, Part II: OARSI evidence-based, expert consensus guidelines. Osteoarthritis and cartilage. 2008;16(2):137–62. 10.1016/j.joca.2007.12.013 18279766

[9] Mafi JN, McCarthy EP, Davis RB, Landon BE. Worsening trends in the management and treatment of back pain. JAMA internal medicine. 2013;173(17):1573–81. 10.1001/jamainternmed.2013.8992 23896698 PMC4381435

[10] Major-Helsloot M, Crous L, Grimmer-Somers K, Louw Q. Management of low back pain at primary care level in South Africa: up to standards? Physiotherapy. 2015;101:e934–e5. 10.4314/ahs.v14i3.28 PMC4209660 25352891

[11] Bishop A, Foster NE, Thomas E, Hay EM. How does the self-reported clinical management of patients with low back pain relate to the attitudes and beliefs of health care practitioners? A survey of UK general practitioners and physiotherapists. PAIN®. 2008;135(1):187–95. 18206309 10.1016/j.pain.2007.11.010 PMC2258319

[12] Darlow B, Fullen BM, Dean S, Hurley DA, Baxter GD, Dowell A. The association between health care professional attitudes and beliefs and the attitudes and beliefs, clinical management, and outcomes of patients with low back pain: a systematic review. European Journal of Pain. 2012;16(1):3–17. 10.1016/j.ejpain.2011.06.006 21719329

[13] Ostelo RW, Vlaeyen JW. Attitudes and beliefs of health care providers: Extending the fear‐avoidance model. Pain. 2008;135(1–2):3–4. 10.1016/j.pain.2007.12.003 18215467

[14] Rainville J, Carlson N, Polatin P, Gatchel RJ, Indahl A. Exploration of physicians’ recommendations for activities in chronic low back pain. Spine. 2000;25(17):2210–20. 10973405 10.1097/00007632-200009010-00012

[15] Bishop A, Thomas E, Foster NE. Health care practitioners’ attitudes and beliefs about low back pain: a systematic search and critical review of available measurement tools. Pain. 2007;132(1):91–101. 17346889 10.1016/j.pain.2007.01.028

[16] Houben R, Ostelo RW, Vlaeyen JW, Wolters PM, Peters M, Berg SG. Health care providers' orientations towards common low back pain predict perceived harmfulness of physical activities and recommendations regarding return to normal activity. European Journal of Pain. 2005;9(2):173–83. 10.1016/j.ejpain.2004.05.002 15737810

[17] Ostelo R, Stomp-van den Berg S, Vlaeyen J, Wolters P, De Vet H. Health care provider's attitudes and beliefs towards chronic low back pain: the development of a questionnaire. Manual therapy. 2003;8(4):214–22. 14559044 10.1016/s1356-689x(03)00013-4

[18] Holden MA, Nicholls EE, Young J, Hay EM, Foster NE. UK‐based physical therapists' attitudes and beliefs regarding exercise and knee osteoarthritis: Findings from a mixed‐methods study. Arthritis Care & Research. 2009;61(11):1511–21. 19877105 10.1002/art.24829

[19] Mutsaers J-H, Pool-Goudzwaard A, Ostelo R, Peters R, Koes B, Verhagen A. The psychometric properties of the PABS-PT in neck pain patients: A validation study. Manual therapy. 2014;19(3):208–14. 10.1016/j.math.2013.12.004 24560002

[20] Vonk F, Pool JJ, Ostelo RW, Verhagen AP. Physiotherapists' treatment approach towards neck pain and the influence of a behavioural graded activity training: an exploratory study. Manual therapy. 2009;14(2):131–7. 10.1016/j.math.2007.12.005 18375173

[21] Mutsaers J-H, Peters R, Pool-Goudzwaard A, Koes B, Verhagen A. Psychometric properties of the Pain Attitudes and Beliefs Scale for Physiotherapists: a systematic review. Manual therapy. 2012;17(3):213–8. 10.1016/j.math.2011.12.010 22277324

[22] Dalkilinc M, Cirak Y, Yilmaz GD, Parlak Demir Y. Validity and reliability of Turkish version of the Pain Attitudes and Beliefs Scale for Physiotherapists. Physiotherapy theory and practice. 2014;31(3):186–93. 10.3109/09593985.2014.986351 25539096

[23] Laekeman M-AL, Sitter H, Basler HD. The Pain Attitudes and Beliefs Scale for Physiotherapists: psychometric properties of the German version. Clinical rehabilitation. 2008;22(6):564–75. 10.1177/0269215508087485 18511536

[24] Watson PJ, Bowey J, Purcell‐Jones G, Gales T. General practitioner sickness absence certification for low back pain is not directly associated with beliefs about back pain. European Journal of Pain. 2008;12(3):314–20. 10.1016/j.ejpain.2007.06.002 17659991

[25] Bishop A. Pain Attitudes and Beliefs Scale (PABS). Journal of physiotherapy. 2010;56(4):279 21213945 10.1016/s1836-9553(10)70014-x

[26] DeMars C. Item response theory: Oxford University Press; 2010.

[27] Embretson SE, Reise SP. Item response theory: Psychology Press; 2013.

[28] De Vet HC, Terwee CB, Mokkink LB, Knol DL. Measurement in medicine: a practical guide: Cambridge University Press; 2011.

[29] Edelen MO, Reeve BB. Applying item response theory (IRT) modeling to questionnaire development, evaluation, and refinement. Quality of Life Research. 2007;16(1):5–18. 17375372 10.1007/s11136-007-9198-0

[30] Petrillo J, Cano SJ, McLeod LD, Coon CD. Using Classical Test Theory, Item Response Theory, and Rasch Measurement Theory to Evaluate Patient-Reported Outcome Measures: A Comparison of Worked Examples. Value in Health. 2015;18(1):25–34. 10.1016/j.jval.2014.10.005 25595231

[31] Reeve BB, Fayers P. Applying item response theory modeling for evaluating questionnaire item and scale properties. Assessing quality of life in clinical trials: methods of practice. 2005;2:55–73.

[32] Little RJ. A test of missing completely at random for multivariate data with missing values. Journal of the American Statistical Association. 1988;83(404):1198–202.

[33] Eekhout I, de Vet HC, Twisk JW, Brand JP, de Boer MR, Heymans MW. Missing data in a multi-item instrument were best handled by multiple imputation at the item score level. Journal of clinical epidemiology. 2014;67(3):335–42. 10.1016/j.jclinepi.2013.09.009 24291505

[34] Sijtsma K, Van der Ark LA. Investigation and treatment of missing item scores in test and questionnaire data. Multivariate Behavioral Research. 2003;38(4):505–28. 10.1207/s15327906mbr3804_4 26777444

[35] van Ginkel JR, Sijtsma K, van der Ark LA, Vermunt JK. Incidence of missing item scores in personality measurement, and simple item-score imputation. Methodology. 2015.

[36] Lenferink A, Effing T, Harvey P, Battersby M, Frith P, van Beurden W, et al Construct validity of the Dutch version of the 12-item Partners in Health Scale: Measuring patient self-management behaviour and knowledge in patients with chronic obstructive pulmonary disease. PloS one. 2016;11(8):e0161595 10.1371/journal.pone.0161595 27564410 PMC5001637

[37] Lorenzo-Seva U, Ferrando PJ. FACTOR: A computer program to fit the exploratory factor analysis model. Behavior research methods. 2006;38(1):88–91. 16817517 10.3758/bf03192753

[38] Van der Ark LA. New developments in Mokken scale analysis in R. Journal of Statistical Software. 2012;48(5):1–27.

[39] Timmerman ME, Lorenzo-Seva U. Dimensionality assessment of ordered polytomous items with parallel analysis. Psychological Methods. 2011;16(2):209 10.1037/a0023353 21500916

[40] Timmerman ME, Lorenzo-Seva U, Ceulemans E. The number of factors problem In: Irwing P, Booth T, Hughes D, editors. The Wiley-Blackwell Handbook of Psychometric Testing. Oxford: Wiley-Blackwell; 2015.

[41] Mokken RJ. A theory and procedure of scale analysis: With applications in political research: Walter de Gruyter; 1971.

[42] Sijtsma K, Molenaar IW. Introduction to nonparametric item response theory: Sage; 2002.

[43] Watson R, van der Ark LA, Lin LC, Fieo R, Deary IJ, Meijer RR. Item response theory: how Mokken scaling can be used in clinical practice. Journal of clinical nursing. 2012;21(19pt20):2736–46. 21883577 10.1111/j.1365-2702.2011.03893.x

[44] van der Ark LA, Sijtsme K. The Effect of Missing Data Imputation on Mokken Scale. New developments in categorical data analysis for the social and behavioral sciences. 2005:147.

[45] Reeve BB, Hays RD, Bjorner JB, Cook KF, Crane PK, Teresi JA, et al Psychometric evaluation and calibration of health-related quality of life item banks: plans for the Patient-Reported Outcomes Measurement Information System (PROMIS). Medical care. 2007;45(5):S22–S31. 17443115 10.1097/01.mlr.0000250483.85507.04

[46] Chalmers RP. mirt: A multidimensional item response theory package for the R environment. Journal of Statistical Software. 2012;48(6):1–29.

[47] Mislevy RJ, Wu PK. Missing responses and IRT ability estimation: Omits, choice, time limits, and adaptive testing. ETS Research Report Series. 1996;1996(2):i–36.

[48] Bozdogan H. Model selection and Akaike's information criterion (AIC): The general theory and its analytical extensions. Psychometrika. 1987;52(3):345–70.

[49] Posada D, Buckley TR. Model selection and model averaging in phylogenetics: advantages of Akaike information criterion and Bayesian approaches over likelihood ratio tests. Systematic biology. 2004;53(5):793–808. 10.1080/10635150490522304 15545256

[50] Kang T, Chen TT. Performance of the Generalized S‐X2 Item Fit Index for Polytomous IRT Models. Journal of Educational Measurement. 2008;45(4):391–406.

[51] Orlando M, Thissen D. Further investigation of the performance of S-X2: An item fit index for use with dichotomous item response theory models. Applied Psychological Measurement. 2003;27(4):289–98.

[52] Reeve BB, Wyrwich KW, Wu AW, Velikova G, Terwee CB, Snyder CF, et al ISOQOL recommends minimum standards for patient-reported outcome measures used in patient-centered outcomes and comparative effectiveness research. Quality of Life Research. 2013;22:1889–905. 10.1007/s11136-012-0344-y 23288613

[53] Sijtsma K. Correcting fallacies in validity, reliability, and classification. International Journal of Testing. 2009;9(3):167–94.

[54] Terwee CB, Bot SD, de Boer MR, van der Windt DA, Knol DL, Dekker J, et al Quality criteria were proposed for measurement properties of health status questionnaires. Journal of clinical epidemiology. 2007;60(1):34–42. 10.1016/j.jclinepi.2006.03.012 17161752

[55] Mokkink LB, Terwee CB, Patrick DL, Alonso J, Stratford PW, Knol DL, et al The COSMIN study reached international consensus on taxonomy, terminology, and definitions of measurement properties for health-related patient-reported outcomes. Journal of clinical epidemiology. 2010;63(7):737–45. 10.1016/j.jclinepi.2010.02.006 20494804

[56] Gatchel RJ, Peng YB, Peters ML, Fuchs PN, Turk DC. The biopsychosocial approach to chronic pain: scientific advances and future directions. Psychological bulletin. 2007;133(4):581 10.1037/0033-2909.133.4.581 17592957

[57] Kamper SJ, Apeldoorn A, Chiarotto A, Smeets R, Ostelo R, Guzman J, et al Multidisciplinary biopsychosocial rehabilitation for chronic low back pain: Cochrane systematic review and meta-analysis. Bmj. 2015;350:h444 10.1136/bmj.h444 25694111 PMC4353283

[58] Pincus T, Kent P, Bronfort G, Loisel P, Pransky G, Hartvigsen J. Twenty-five years with the biopsychosocial model of low back pain—is it time to celebrate? A report from the twelfth international forum for primary care research on low back pain. Spine. 2013;38(24):2118–23. 10.1097/BRS.0b013e3182a8c5d6 23970112

[59] Waddell G. 1987 Volvo Award in Clinical Sciences: a new clinical model for the treatment of low-back pain. Spine. 1987;12(7):632–44. 2961080 10.1097/00007632-198709000-00002

