# Supplementary material for: Item response theory evaluation of the biomedical scale of the Pain Attitudes and Beliefs Scale
Source: PLoS One. 2018 Sep 12;13(9):e0202539. doi: 10.1371/journal.pone.0202539 (PMC6135359; doi:10.1371/journal.pone.0202539)
Supplement: S1 Table — (DOCX) [file pone.0202539.s001.docx]

**S1 Table.**

**Polychoric correlation matrix of the biomedical scale of the Pain Attitudes and Beliefs Scale (PABS) in the BeBack data (n = 1016).**

|  | **Item 1** | **Item 2** | **Item 3** | **Item 4** | **Item 5** | **Item 6** | **Item 7** | **Item 8** | **Item 9** | **Item 10** |
| --- | --- | --- | --- | --- | --- | --- | --- | --- | --- | --- |
| **Item 1** | 1.000 |  |  |  |  |  |  |  |  |  |
| **Item 2** | 0.247 | 1.000 |  |  |  |  |  |  |  |  |
| **Item 3** | 0.458 | 0.223 | 1.000 |  |  |  |  |  |  |  |
| **Item 4** | 0.196 | 0.215 | 0.193 | 1.000 |  |  |  |  |  |  |
| **Item 5** | 0.131 | 0.133 | 0.150 | 0.207 | 1.000 |  |  |  |  |  |
| **Item 6** | 0.273 | 0.306 | 0.211 | 0.269 | 0.352 | 1.000 |  |  |  |  |
| **Item 7** | 0.408 | 0.323 | 0.395 | 0.270 | 0.300 | 0.469 | 1.000 |  |  |  |
| **Item 8** | 0.298 | 0.429 | 0.320 | 0.328 | 0.252 | 0.351 | 0.595 | 1.000 |  |  |
| **Item 9** | 0.385 | 0.314 | 0.385 | 0.215 | 0.195 | 0.354 | 0.565 | 0.497 | 1.000 |  |
| **Item 10** | 0.171 | 0.171 | 0.256 | 0.178 | 0.228 | 0.233 | 0.245 | 0.281 | 0.216 | 1.000 |
