# Supplementary material for: Item response theory evaluation of the biomedical scale of the Pain Attitudes and Beliefs Scale
Source: PLoS One. 2018 Sep 12;13(9):e0202539. doi: 10.1371/journal.pone.0202539 (PMC6135359; doi:10.1371/journal.pone.0202539)
Supplement: S2 Table — (DOCX) [file pone.0202539.s002.docx]

**S2 Table**

**Polychoric correlation matrix of the biomedical scale of the Pain Attitudes and Beliefs Scale (PABS) in the DABS data (n = 958)**

|  | **Item 1** | **Item 2** | **Item 3** | **Item 4** | **Item 5** | **Item 6** | **Item 7** | **Item 8** | **Item 9** | **Item 10** |
| --- | --- | --- | --- | --- | --- | --- | --- | --- | --- | --- |
| **Item 1** | 1.000 |  |  |  |  |  |  |  |  |  |
| **Item 2** | 0.342 | 1.000 |  |  |  |  |  |  |  |  |
| **Item 3** | 0.522 | 0.312 | 1.000 |  |  |  |  |  |  |  |
| **Item 4** | 0.180 | 0.317 | 0.118 | 1.000 |  |  |  |  |  |  |
| **Item 5** | 0.173 | 0.200 | 0.157 | 0.169 | 1.000 |  |  |  |  |  |
| **Item 6** | 0.257 | 0.233 | 0.205 | 0.211 | 0.257 | 1.000 |  |  |  |  |
| **Item 7** | 0.534 | 0.395 | 0.452 | 0.239 | 0.270 | 0.317 | 1.000 |  |  |  |
| **Item 8** | 0.396 | 0.485 | 0.356 | 0.269 | 0.168 | 0.216 | 0.574 | 1.000 |  |  |
| **Item 9** | 0.468 | 0.367 | 0.439 | 0.185 | 0.229 | 0.215 | 0.567 | 0.542 | 1.000 |  |
| **Item 10** | 0.052 | 0.176 | 0.134 | 0.114 | 0.267 | 0.251 | 0.199 | 0.150 | 0.248 | 1.000 |
